# Long-Term Monitoring of Noxious Bacteria for Construction of Assurance Management System of Water Resources in Natural Status of the Republic of Korea

**DOI:** 10.4014/jmb.2004.04064

**Published:** 2020-07-31

**Authors:** Young Yil Bahk, Hyun Sook Kim, Ok-Jae Rhee, Kyung-A You, Kyung Seon Bae, Woojoo Lee, Tong-Soo Kim, Sang-Seob Lee

**Affiliations:** 1 Department of Biotechnology, College of Biomedical and Health Science, Konkuk University, Chungju 27478, Republic of Korea; 2 Department of Life Science, Graduate School, Kyonggi University, Suwon 167, Republic of Korea; 3 DK EcoV Environmental Microbiology Lab., Cheonan 1075, Republic of Korea; 4 Environmental Infrastructure Research Department, Water Supply and Sewerage Research Division, National Institute of Environmental Research, Incheon 22689, Republic of Korea; 5 Department of Public Health Science, Graduate School of Public Health, Seoul National University, Seoul 08826, Republic of Korea; 6 Department of Parasitology and Tropical Medicine, School of Medicine, Inha University, Incheon 22212, Republic of Korea

**Keywords:** Noxious bacteria, catchment-scale investigation, water resource, climate change

## Abstract

Climate change is expected to affect not only availability and quality of water, the valuable resource of human life on Earth, but also ultimately public health issue. A six-year monitoring (total 20 times) of *Escherichia coli* O157, *Salmonella enterica*, *Legionella pneumophila*, *Shigella sonnei*, *Campylobacter jejuni*, and *Vibrio cholerae* was conducted at five raw water sampling sites including two lakes, Hyundo region (Geum River) and two locations near Water Intake Plants of Han River (Guui region) and Nakdong River (Moolgeum region). A total 100 samples of 40 L water were tested. Most of the targeted bacteria were found in 77% of the samples and at least one of the target bacteria was detected (65%). Among all the detected bacteria, *E. coli* O157 were the most prevalent with a detection frequency of 22%, while *S. sonnei* was the least prevalent with a detection frequency of 2%. Nearly all the bacteria (except for *S. sonnei*) were present in samples from Lake Soyang, Lake Juam, and the Moolgeum region in Nakdong River, while *C. jejuni* was detected in those from the Guui region in Han River. During the six-year sampling period, individual targeted noxious bacteria in water samples exhibited seasonal patterns in their occurrence that were different from the indicator bacteria levels in the water samples. The fact that they were detected in the five Korea’s representative water environments make it necessary to establish the chemical and biological analysis for noxious bacteria and sophisticated management systems in response to climate change.

## Introduction

Life on Earth are greatly affected by the dynamics of climate system, especially the Earth’s surface climate. In particular, infectious pathogens are emerging as a source of issue as many aspects of public health accompanying the climate change are widely recognized [1, 2]. The term pathogen covers a wide range of disease agents, such as virus, bacteria, parasitic germs, and fungi that can affect human beings either directly or indirectly through influencing the habitat, environment, or by competing with other pathogens. Climate change is a global phenomenon and is expected to accelerate in the future, especially in situations where the extent of climate change on Korean peninsula is relatively large (*e.g.*, temperature rise, rainfall change, *etc*.) [3]. The annual mean temperature has been increasing at a rate of 0.52°C per decade and is significantly larger over urbanized areas [4], and it is anticipated that the incidence and geographic distribution of vector-borne diseases will change as a result [5].

*Shigella* is a genus of gram-negative pathogenic enterobacteria and a pathogenic variant of *Eschericha coli* comprising four groups, *Shigella boydii*, *S. dysenteriae*, *S. sonnei*, and *S. flexneri* [6]. *Shigella* species are waterborne and food-borne agents of bacillary gastrointestinal dysentery or shigellosis responsible for an estimated 80-165 million cases worldwide and account for a primary cause of childhood morbidity and mortality [7]. *S. sonnei* and *S. flexneri* result in most Shigellosis cases, with *S. sonnei* causing over 80% of all shigellosis infections and is increasingly found in developing countries [8]. In developing countries, especially where exist various public health problem caused by poor hygiene standards, a safe supply of drinking water influences the risk of public health.

Enterohemorrhagic *E. coli* O157 is a subtype of shiga toxin-producing *E. coli* and a primary food-borne pathogen causing the severe diseases in human such as hemolytic uremic syndrome, thrombotic thrombocytopenic purpura, and hemorrhagic colitis worldwide, although elderly and children are more expugnable [9].

*Salmonellae* are facultative anaerobic gram-negative bacteria belonging to the family Enterobacteriaceae and are a medically pivotal pathogen; two main species are *Salmonella bongorin* and *S. enterica* [10]. *S. enterica* has six subspecies that are composed of over 1500 subtypes some of which have profound medical significance [11]. Salmonella is an international food-borne intravacuolar pathogen causing a huge number of deaths and has a substantial cost burden. *S. enterica* subsp. enterica is responsible of more than 99% of human salmonellosis cases [12].

*Legionella pneumophila*, which causes community-acquired pneumonia that requires hospitalization, is an opportunistic pathogen that is omnipresent in aquatic environments in which it replicates in free-living amoebae [13, 14]. *L. pneumophila* pneumonia is strongly associated with high morbidity. Moreover, legionellosis is consistently reported as one of the top three most identified respiratory pathogens in community-acquired pneumonia, along with the hospital-acquired pneumonia [15].

Campylobacterota, formerly identified as Epsilon proteobacteria, are a whole bunch of gram-negative motile bacteria found in manifold ecological habitats [16]. *Campylobacter*ota are pivotal clinical pathogens in human; the gut of half of the human beings is mass-dwelled with the stomach ulcer-causing bacterium *Helicobacter pylori*, while *Campylobacter jejuni* is a ubiquitous gastrointestinal pathogen and one of the majorly diagnosed bacterial food-borne pathogens in human and remains among the most common causes of bacterial gastroenteritis in many areas of world [17]. *Campylobacter*ia infection causes the development of miscarriage, septicemia, gastroenteritis, proctitis, meningitis, and many neurological diseases, the foremost of which is Guillain-Barré syndrome among other central nervous system (CNS) diseases with similar acute progresses. Large global population (1-10% of the whole) can influence the risk of campylobacteriosis annually [18]. The non-food-borne transmission pathways for *Campylobacter* to human are birds and animals in which *C. jejuni* is part of normal flora, with the major pathway of transmission being the ingestion of contaminated food or drinking water [19].

*Vibrio cholerae* is a motile, aquatic curved-rod facultative gram-negative anaerobe belonging to the family of *Vibrionaceae*. Cholera caused by an etiological agent, *V. cholerae*, has been a serious epidemic secretary diarrheal disease that can quickly lead to severe dehydration and prove fatal within hours if untreated. Strains of *V. cholerae* inhabit both marine and freshwater ecosystems [20, 21]. Despite great betterments in hygiene, water quality, and sanitation, as well as in the clinical treatment, the disease is still estimated to cause about 100,000 deaths every year.

Climate change is causing water scarcity not only through increased temperatures and prolonged drought times but also through the degradation of water resources caused by increasing levels of pathogens and other contaminants posing significant health risks [22]. Thus, there is clearly a strong need for establishing management strategies and constant monitoring the water resources based on the results of testing water for contamination from relevant sources. Herein, we report the results on long-term (six-year) surveillance of noxious bacteria (*E. coli* O157, *S. enterica*, *L. pneumophila*, *S. sonnei*, *C. jejuni*, and *V. cholerae*) from August 2013 to February 2019 at various locations in Republic of Korea (Korea) to help the establishment of the management systems to maintain water quality and security.

## Material and Methods

### Collection Sites

A catchment scale investigation of the prevalence of *E. coli* O157, *S. enterica*, *L. pneumophila*, *S. sonnei*, *C. jejuni*, and *V. cholerae* was carried out. Water samples were collected 20 times from August 2013 to February 2019 from five surface water sampling locations: two lakes (Lake Soyang in Gangwon province and Lake Juam in Jeollanam province), Hyundo region (near Hyundo Bridge of Geum River at Shintanjin-dong in Daejeon Metropolitan City (Daejeon)), and two water intake plants (the Guui region on Han River in Seoul Special City (Seoul) and the Moolgeum region on Nakdong River in Gimhae-si) (Table 1). These five locations were selected to reflect the environment in response to changes in the landscape according to the climate change scenario by Intergovernmental Panel on Climate Change (IPCC): the Moolgeum region of Nakdong River, which is a subtropical zone; Lake Juam, which is classified as Representative Concentration Pathway (RCP) 4.5 proceeding to subtropical zone; the Guui region of Han River and the Hyundo region of Geum River, which are RCP 8.5 zones; and Lake Soyang, which is considered as non-subtropical zone in Korea.

### Testing of Indicator Bacteria

Water samples were collected from each location. *E. coli* contamination were measured in samples using a Most Probable Number (MPN) assay. One hundred ml aliquots of water samples from each location were evaluated for total coliform (TC), fecal coliform (FC) and *E. coli* contamination and an IDEXX Colilert-18 and Quanti-Tray System (IDEXX Laboratories, USA). The collected water samples were placed immediately in a refrigerator (4°C) upon collection using sterile bags and transported to private laboratory in Kyonggi University for further processing. Briefly, the process started by adding a Colilert-18 reagent to each sample until it fully dissolved. The mixture was placed in a Quanti-Tray, which was sealed and incubated at 35°C and 44.5°C for 24 h each. Following incubation and positive well counts, the results were obtained using the IDEXX results table, where the number of colored and fluorescing large and small cells determined the MPN for coliform bacteria and *E. coli*. For each test sample, appropriate dilutions were prepared. This system is based on the MPN technique [23] and is a semiautomatic enzyme-based assay reduced to multi-wells. Control samples of commercially available sterile water were included along with the samples to evaluate cross- contamination.

### Quality of the Collected Water Samples

Physiochemical parameters such as *p*H, total dissolved solids, dissolved oxygen (DO), total nitrogen, ammonia, nitrate, nitrite, phosphate, and sulfate were analyzed according to the Standard Methods for the Examination of Water and Wastewater [24]. Turbidity and conductivity were measured with a HACH 1900C portable turbidity meter (HACH, USA) and a HACH sension 5 conductivity meter (HACH), respectively. *p*H was measured on-site using individually calibrated portable testers. Chemical oxygen demand (COD) and ammonium content (NH_3_-N) were measured according to standard method [25, 26].

### Sample Collection and Analysis

The targeted bacteria were *S. sonnei*, *E. coli* O157, *S. enterica* spp., *L. pneumophila*, *C. jejuni*, and *V. cholerae*. Spatially distributed samples were aseptically collected at five locations using sterile containers (Table 1). Samples were simultaneously and in parallel examined for the detection of 6 noxious bacteria. One-liter aliquots from each of five consecutive sampling were filtered using 0.2 mm filters to collect particulates. The filters were processed and extracted DNA using GeneAll Exgene Soil DNA kits (GeneAll Biotechnology, Korea) according to the manufacturer’s recommendation. The concentration of the extracted DNA was determined by measuring ultraviolet absorbance at 260 nm using a spectrophotometer (NanoDrop ND-1000, Thermo Fisher Scientific, USA), after which the samples were stored at -70°C before use.

Real-time PCR analysis was conducted with 10 ul of SYBR green master mix (Thermo Fisher Scientific) and 10 pM specific primer sets in a reaction volume of 20 ul using CFX96 Real-time PCR system (Bio-Rad Laboratories, USA). The primer set sequences and reaction conditions for each targeted noxious bacterium and the amplified target sizes represent in Table 2. The specificity of the primers was confirmed using a BLAST search in GenBank database from NCBI. For each bacterium tested in this monitoring, the BLASTn searches yield no solid match to any of the other identified bacterium reference sequences. Specificity tests were performed using conventional PCR techniques for each species or subspecies primer set against DNA samples from various bacterium strains [27]. Matches between the cyclic quantification (Cq) value and each of noxious bacteria detection was verified, and positive estimation was determined for a single peak using the Cq value. The positive samples were analyzed and confirmed by sequencing the 16s rDNA fragments by Macrogen Inc. (Korea). Analysis of the derived nucleotide sequences was performed for matching genotypes using the NCBI-BLAST service to target the noxious bacteria.

## Results

### Physicochemical Parameters in the Water Samples

There were slight variations in physiochemical parameters among the water sample collecting sites. The water temperature at the Guui region and Lake Juam tended to increase slightly, while no definite trend was observed for precipitation (Data not shown). The other collection sites did not show a definite tendency in the parameters of precipitation or water temperature. Table 3 summarizes the physiochemical parameters of water samples from the five sample collecting sites during the periods from August 2013 to February 2019. All the parameters other than precipitation and water temperature fluctuated continuously through the year. The *p*H values are in the range of 6.5 to 8.5 (in a descending order: Guui region > Moolgeum region > Hyundo region > Lake Soyang > Lake Juam), which according to the World Health Organization (WHO) guidelines for drinking water [28], the *p*H values at the surface fall within the normal limit. Most of the monitoring locations had a sufficient DO level (more than 7 mg/l), although one was borderline (Lake Juam). The conductivity values of the collected water samples were ranged from 70 to 350 mS/cm on average, which are well within the unpolluted freshwater range of 10 to 1,000 mS/cm. The average amount of total nitrogen in the water samples from the collection sites was 1.898 ± 0.850 mg/l, while ammoniacal nitrogen (NH_3_ or NH_4_^+^) did not consistently exceed 0.3 mg/l. The acceptable amount of nitrates in drinking water is up to around 44 mg/l [29], so the samples from the locations were well within this (0.4-2.1 mg/l). The phosphate level in the samples was 0.025 ± 0.021 mg/l, which is well within the WHO guideline of 1 mg/l. In general, the Moolgeum region on Nakdong River had the highest values for BOD, COD, conductivity, total nitrogen, total phosphorus, and phosphate, while the Guui region on Han River in Seoul had highest values for *p*H, DO, ammonia, and nitrate.

### Indicator Bacteria and Water-Quality Monitoring Stations

The monitoring points in this study were selected for the water quality measurements due to the needs for long-term monitoring and management of Korean rivers and lakes and the links between the nearby water quality measuring network points according to the prediction scenario for climate change: Lake Soyang in the exceptional subtropical zone and the Guui region on the Han River in Seoul, Hyundo region (near Hyundo Bridge of Geum River at Shintanjin-dong in Daejeon), Lake Juam, and the Moolgeum region on Nakdong River in the subtropical zone.

In practice, it is impossible to enumerate all pathogens in water-source because of the absence of specific detection techniques. Thus, indicator bacteria including TC, FC and *E. coli* are traditionally used to indicate the presence of a pathogens, especially in wastewater as well as other intestinal pathogens [30]. The presence of TC and FC is indicative of human fecal contamination. TC, FC, and/or *E. coli* were detected in almost samples collected across 6 year monitoring and 23% of the samples exceeded the regulations provided by the Pennsylvania Department of Environmental Protection (PA DEP) form TC (5,000 CFU/100 ml) at concentration ranging from 0 to 1.9 × 10^9^ MPN/100 ml (CFU and MPU are equivalent), which were mainly observed between August and October [31]. Concentrations of the FC in 13% exceeded the PA DEP regulations for fecal coliforms (200 CFU/ 100 ml) during the investigation period [31]. Spatially, the Guui region of Han River was the highest contaminated place among the monitored sites in this study, followed by Moolgeum region of Nakdong River. During the period under investigation, the TC (average: 1.2 × 10^4^ MPN/100 ml) rather than FC (average: 563 MPN/100 ml) or *E. coli* (average: 313 MPN/100 ml) were detected highest. The averages of three indicator bacteria in August 2017 were highest (3.8 × 10^4^, 1.0 × 10^4^, and 5.8 × 10^3^ MPN/100 ml for TC, FC and *E. coli*, respectively) (Table 4). Lake Soyang and Lake Juam had lower contamination than the other sites in this study. However, it is not evident from the information on precipitation and physicochemical characteristics whether the collected indicator bacteria in the collected samples were higher in August 2017 compared to other collection periods. Overall, the distribution of the indicator bacteria was found to be the highest in August (when the water temperature was high), followed by October.

### Relationship between Physicochemical Parameters and Indicator Bacteria

Physicochemical parameters are among the major factors involved in the management and mitigation of non-point source pollution, and the effect of fecal contamination on the quality of water is a matter of quite concern. Information gained through the regular monitoring of water quality allows estimation of the likelihood pathogens-related waterborne disease [32]. In general, indicator bacteria (TC, FC, and *E. coli*) tended to decrease over time except in 2013 (Table 4). TC levels were higher in August and October of the year and remained at a certain level depending on the time of collection. However, TC levels in Lake Soyang and Lake Juam were the highest in October when the water temperature was low. The detection rates of FC and *E. coli* were stable throughout the monitoring periods. Finally, it is evident that the distribution of indicator bacteria was related to changes in turbidity due to water temperature and precipitation.

### Detection of Noxious Bacteria in Monitoring Sites

**Fig. 1:**
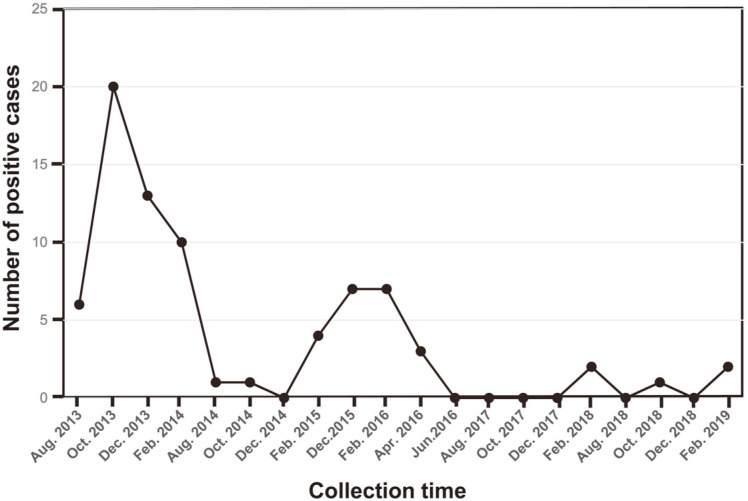
Total positive incidence cases of noxious bacteria surveillance in this study during the monitoring period.

**Fig. 2:**
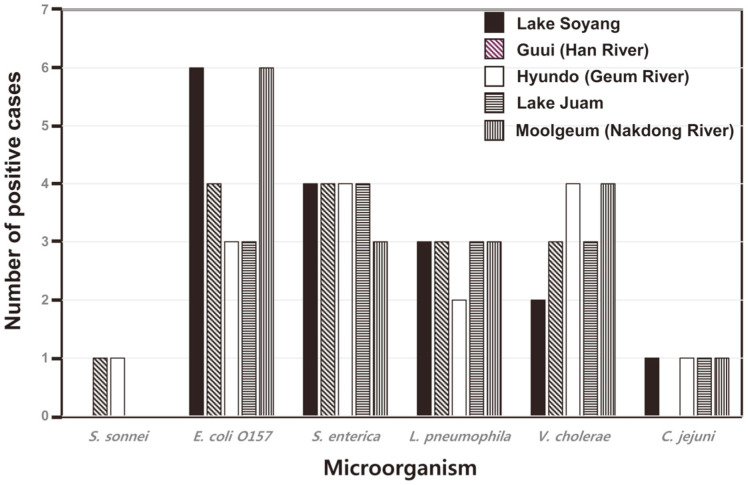
Positive incidence cases of each noxious bacterium at each collecting site.

Most of the targeted bacteria were found in 77% of the samples and at least one of the target bacteria was detected (65%) (Fig. 1, Table 5). Among all the detected bacteria, *E. coli* O157 were the most prevalent with a detection frequency of 22% (22/100), while *S. sonnei* was the least prevalent with a detection frequency of 2% (2/ 100 samples). Nearly all of the bacteria (except for *S. sonnei*) were present in samples from Lake Soyang, Lake Juam, and Nakdong River (Fig. 2), while *C. jejuni* was detected in those from Han River. During the six-year sampling period, individual targeted noxious bacteria in water samples exhibited seasonal patterns in their occurrence that were different from the indicator bacteria levels in the water samples. The occurrence of noxious bacteria in the samples was higher during the colder months (October, December, and February) than the warmer ones. However, after April 2016, the occurrences of noxious bacteria in the water samples dramatically decreased to 10.39%. This can be attributed to the authorities’ effort, such as sewage system management, to improve the water quality. Detection of TC, FC and *E. coli* in the water samples could not predict the total noxious bacteria presence.

### Statistical Analysis of Correlation between Indicator Bacteria and the Tested Noxious Bacteria

We performed correlation tests between the monitored six noxious bacteria and the tested three indicator bacteria (TC, FC, *E. coli*) using permutation technique [33]. Testing results are summarized in Table 6. Only between *S. enterica* and TC has *p*-value less than 0.05. All the other relationships were not able to look at a significant association. Even in the case of the association between *S. enterica* and TC, when their *p*-value were adjusted by the Bonferroni calibration, it did not produce any significant results. After Bonferroni adjustment, all the combinations show *p*-values larger than 0.05. Thus, we concluded that the current results show no statistically significant association in any combination.

## Discussion

The worldwide burden of infectious waterborne disease is considerable, and the bacterial pathogens are strongly resistant in the water environment and to most disinfectants. Some bacterial agents such as *S. sonnei*, *C. jejuni* and *E. coli* O157 can contaminate pristine waters through wildlife and human activities. In addition, climate variables such as precipitation, temperature that have changed significantly as a result of global climate change are major driving forces of food- and waterborne diseases and alter the exposure pathways. These determinants could influence the fate and transport of pathogens, as well as their stability, reproduction rates, and viability in the environment. Therefore, sophisticated and consistent surveillance systems and means should be put in place to monitor the targeted pathogen candidates for serious waterborne diseases.

Some of the noxious bacteria exhibited spatial and seasonal patterns at the collecting sites in this study. The presence of *C. jejuni* in samples from four of the targeted sampling collection sites (except the Guui region of Han River) indicates that positive cases are in fall and winter (October, December and February) but not in spring and summer seasons (August, April, and June), which coincides with previously reported studies [34, 35]. In addition, the positive cases for *L. pneumophila* are in winter except for one in August 2013 at the Moolgeum region site. In the case of *S. sonnei*, there were only two positive cases of samples from the Guui region and the Hyundo region in October 2013 were not linked to seasonality. Similarly, the indicator bacteria TC, FC and *E. coli* were not consistently and significantly correlated with the detection of the targeted noxious bacteria (Table 6). These data indicate that indicator bacteria and physiochemical parameters used in this study are not potential candidates for predicting the presence of typical noxious bacteria such as *S. sonnei*, *E. coli* O157, *S. enterica* spp., *L. pneumophila*, *C. jejuni*, and *V. cholerae* in the surface water at the five targeted surface water sampling locations including the Guui region, the Moolgeum region, the Hyundo region, Lake Soyang and Lake Juam.

According to the results of the monitoring in this study, the occurrences of noxious bacteria in water samples were dramatically decreased after April 2016. Although it is difficult to elucidate the specific cause, this could be attributed to the authorities’ effort, such as sewerage system management and social good-informed cognition, to improve the water quality. Korea achieved 92.1% penetration rate of sewage into the advanced countries through the first National Sewage Comprehensive Plan (NSCP) (2007-2015) through continuous expansion of sewage treatment facilities and sewage systems, improved sewage maintenance, enhancement of sewerage and sewerage management, establishment of water resource circulation utilization systems, and improved sewage treatment technology and sewage sludge treatment [36].

This study has a critical limitation. First of all, in some years during the study, the collection of surface water samples has limitations that have not been carried out as originally planned and thus we were not able to proceed with consistent sample collection and monitoring during the summer season. In addition, since this study was only based on the genetic analysis using PCR methods, we were not able to determine the infectivity and pathogenesis despite the positive detection. Nevertheless, this study was designed and practiced at these specific sites as a project of the National Institute of Environmental Research funded by the Ministry of Environment of the Republic of Korea. In fact, despite the growing interest in monitoring noxious microorganisms, it is difficult to find a case of research on their distribution and monitoring related to climate change at Korea or abroad. The Ministry of Environment of the Republic of Korea recognized the need for this research to provide public health security and secure drinking water stability because of water temperature rise, flooding, drought and heat waves due to climate change increase the prevalence of noxious microbes. The Ministry of Environment had set a Priority Management List (PML) of 20 noxious microbes in groups including TC bacteria, FC bacteria, pathogenic *E. coli*, enterococci, fecal *Streptococci*, *Pseudomonas* as concerns about unregulated waterborne microbes increase.

In conclusion, it was not possible to determine the infectivity and pathogenicity on the six noxious bacteria examined in this study, and it was difficult to precisely identify any noticeable seasonal or regional effects. However, the fact that they were detected in the five Korea’s representative water environments comprising lakes, rivers, and drinking water collecting sites make it necessary to establish the chemical and biological analysis for noxious bacteria and sophisticated management systems in response to climate change. Thus, relying on predictive models and monitoring for timely warning can protect the health of the public.

## Figures and Tables

**Table 1:** Climate characteristics and geographic indexes the sample collection sites.

Collection sites	Climate classification	Geographic indexes	Characteristics
Lake Soyang	Exceptional Subtropic Zone	37.5654/127.4855	Lake
The Guui region on River Han in Seoul	RCP 8.5 Subtropic Zone	37.3305/127.0641	River
The Hyundo region (near Hyundo Bridge of Geum River at Shintanjindong in Daejeon)	RCP 8.5 Subtropic Zone	36.2724/127.2544	River
Lake Juam	RCP 4.5 Subtropic Zone	35.0340/127.1412	Lake
The Moolgeum Region on the Nakdong River	Subtropic Zone	35.1834/128.5837	River

**Table 2:** Primer set sequences used for noxious bacteria and cycling parameters in this study.

Species	Target gene	Primer sequences	Product size	Cycling parameters
*Shigella sonnei*	Hypothetical protein	F: 5’-ACGCGTTAAAGATGATGCCTGTT-3’	325 bp	Initial denaturation: 95°C (2.5 min) 45 cycles of 95°C (10 sec), 60°C (20 sec) Denaturation: 95°C (10 sec) Slope range: 65-95°C for melting curve and melting peak
		R: 5’-TGCCGCTAAAATCCTTCTGTCCT-3’		
*E. coli* O157	Hypothetical protein	F: 5-GCCGTACATGCTGCTGAGAGTC-3’	215 bp	Initial denaturation: 95°C (2.5 min) 45 cycles of 95°C (10 sec), 59°C (20 sec) Denaturation: 95°C (10 sec) Slope range: 65-95 °C for melting curve and melting peak
		R: 5’-TAGCCCCATATAGCGTAAGAAT-3’		
*Salmonella enterica*	Hypothetical protein	F: 5’-CGCGTCGCTTCGTTCTGTATCAT-3’	353 bp	Initial denaturation: 95°C (2.5 min) 45 cycles of 95°C (10 sec), 50°C (20 sec) Denaturation: 95°C (10 sec) Slope range: 65-95 °C for melting curve and melting peak
		R: 5’-GCGCTGCCACTCTCGGTTTCTTAT-3’		
*Legionella pneumophila*	Hypothetical protein	F: 5’-ACACGTTGAAGAGGAGTTAG-3’	264 bp	Initial denaturation: 95°C (2.5 min) 45 cycles of 95°C (10 sec), 59°C (20 sec) Denaturation: 95°C (10 sec) Slope range: 65-95 °C for melting curve and melting peak
		R: 5’-ACAAGCTCTACTTCAATGCC-3’		
*Vibrio cholerae*	Hypothetical protein	F: 5’-CCGTTGAGGCGAGTTTGGTGAGA-3’	195 bp	Initial denaturation: 95°C (2.5 min) 45 cycles of 95°C (10 sec), 52°C (20 sec) Denaturation: 95°C (10 sec) Slope range: 65-95 °C for melting curve and melting peak
		R: 5’-GTGCGCGGGTGGAAACTTATGAT-3’		
*Campylobacter jejuni*	Hypothetical protein	F: 5’-AAAAAGAGATTTATATTAACAAAA-3’	177 bp	Initial denaturation: 95°C (2.5 min) 45 cycles of 95°C (10 sec), 55°C (20 sec) Denaturation: 95°C (10 sec) Slope range: 65-95 °C for melting curve and melting peak
		R: 5’-GCTTAATTGTATAGTTTATATTATC-3’		

**Table 3:** Physicochemical characteristics of the water samples of the five collected sites in this study.

Parameter Sites	Temp. (°C)	pH	Dissolved oxygen (mg/l)	BOD (mg/l)	COD (mg/l)	Conductivity (mS/cm at 20°C)	Total nitrogen (mg/l)	Ammonia (mg/l)	Nitrate (mg/l)	Total phosphorus (mg/l)	Phosphate (mg/l)
The Guui region on the Han River in Seoul	12.71±9.11	8.125±0.17	12.24±1.89	1.515±0.51	4.115±0.61	209.3±47.96	2.684±0.47	0.097±0.091	2.028±0.33	0.038±0.019	0.0065±0.008
The Moolgeum region on the Kakdong River	15.06±9.50	8.075±0.37	10.925±2.45	1.955±0.52	6.265±1.22	336.25±104.24	2.742±0.57	0.086±0.035	1.959±0.60	0.047±0.027	0.0112±0.013
The Hyundo region (near Hyundo Bridge of Geum River in Daejeon)	13.26±6.85	7.905±0.23	10.335±2.50	0.73±0.25	3.875±0.61	167.35±26.31	1.479±0.25	0.106±0.273	1.088±0.34	0.015±0.00	0.0032±0.005
Lake Soyang	9.45±4.70	7.33±0.35	8.395±1.65	1.18±0.26	2.09±0.32	76.2±5.73	1.867±0.31	0.024±0.014	1.377±0.17	0.011±0.006	0.0025±0.002
Lake Juam	11.93±5.40	6.82±0.37	6.985±3.14	0.82±0.19	2.94±0.47	79.6±8.08	0.718±0.11	0.069±0.062	0.472±0.12	0.132±0.005	0.0033±0.003

**Table 4:** Total indicator bacteria (MPN/100 ml) including TC, FC, and *E. coli* during the monitoring period.

Indicator bacteria	Collection sites	Aug, 2013	Oct, 2013	Dec 2013	Feb, 2014	Aug, 2014	Oct, 2014	Dec, 2014	Feb, 2015	Dec, 2015	Feb, 2016	Apr, 2016	Jun, 2016	Aug, 2017	Oct, 2017	Dec, 2017	Feb, 2018	Aug, 2018	Oct, 2018	Dec, 2018	Feb, 2019	Average
Total Coliform (TC)	Lake Soyang	90,000	12,000	5,400	100	610	2,400	550	67	32	70	150	1,270	3,700	1,300	160	7	0	3,400	170	65	6,072.55
	Han River	190,0000	85,000	5,500	200	5,200	1,200	370	130	1,700	1,400	480	1,100	160,00	1,700	2,900	4,400	14,000	1,200	610	300	23,869.5
	Geum River	63,000	11,000	5,800	1,000	1,100	37	2,000	140	290	1,300	17,000	2,500	24,000	22,000	650	410	0	34,000	2,900	770	9,494.85
	Lake Juam	110,000	5,900	5,400	1,400	6,000	5,500	130	14	150	17	260	980	7	2,400	20	0	1,000	5,200	390	16	7,239.2
	Nakdong River	190,000	48,000	1,900	100	2,300	9,200	460	56	12	240	1,900	3,500	3,700	8,700	1,000	220	6,900	3,900	520	180	14,139.4
Fecal Coliform (FC)	Lake Soyang	1	1	0	0	1	5.2	1	0	0	0	2	0	5	73	4	1	0	1	2	1	4.91
	Han River	13	37	40	12	30	120	25	29	79	32	38	200	48,000	290	96	820	650	54	30	11	2530.3
	Geum River	51	2	5.1	119	0	6	0	3	3	670	31	290	2,100	96	22	9	0	150	120	1	183.905
	Lake Juam	1	5.2	3.1	0	0	310	2	0	0	0	8	0	0	68	2	0	200	3	0	0	30.115
	Nakdong River	4.1	1	1	0	5.2	310	6.3	17	1	0	9	6	32	610	37	4	220	35	3	2	65.18
*E. coli*	Lake Soyang	1	1	0	0	0	4.1	1	0	0	0	0	1	0	0	0	0	0	0	0	0	0.405
	Han River	8.5	20	38	8.6	30	73	6.3	5	71	32	23	96	29,000	100	28	820	20	33	23	9	1,522.22
	Geum River	0	1	5.1	1	10	0	15	0	3	3	19	23	210	42	28	9	0	120	96	3	29.405
	Lake Juam	1	3	3.1	0	0	1	0	0	0	0	4.1	0	0	0	0	0	0	0	0	0	0.61
	Nakdong River	2	1	1	0	4.1	240	0	3	0	0	6	5	11	5	0	1	0	14	0	1	14.705

**Table 5:** Positive incidence cases of each noxious bacterium at the five-water sample collecting sites during the monitoring period in this study.

		Aug. 2013	Oct. 2013	Dec. 2013	Feb. 2014	Aug. 2014	Oct. 2014	Dec. 2014	Feb. 2015	Dec. 2015	Feb. 2016	Apr. 2016	Jun. 2016	Aug. 2017	Oct. 2017	Dec. 2017	Feb. 2018	Aug. 2018	Oct. 2018	Dec. 2018	Feb. 2019	Total
Lake Soyang	*S. sonnei*																					0
	*E. coli* O157	Posi.	Posi.	Posi.	Posi.					Posi.							Posi.					6
	*S. enterica*	Posi.	Posi.	Posi.						Posi.												4
	*L. pneumophila*		Posi.						Posi.		Posi.											3
	*V. cholerae*								Posi.	Posi.												2
	*C. jejuni*									Posi.												1
Han River	*S. sonnei*		Posi.																			1
	*E. coli* O157	Posi.	Posi.	Posi.							Posi.											4
	*S. enterica*	Posi.	Posi.	Posi.						Posi.												4
	*L. pneumophila*		Posi.			Posi.					Posi.											3
	*V. cholerae*		Posi.		Posi.						Posi.											3
	*C. jejuni*																					0
Geum River	*S. sonnei*		Posi.																			1
	*E. coli* O157		Posi.	Posi.	Posi.																	3
	*S. enterica*		Posi.	Posi.	Posi.						Posi.											4
	*L. pneumophila*		Posi.		Posi.																	2
	*V. cholerae*		Posi.	Posi.	Posi.																Posi.	4
	*C. jejuni*		Posi.																			1
Lake Juam	*S. sonnei*																					0
	*E. coli* O157		Posi.	Posi.	Posi.																	3
	*S. enterica*	Posi.			Posi.					Posi.	Posi.											4
	*L. pneumophila*						Posi.		Posi.										Posi.			3
	*V. cholerae*			Posi.					Posi.			Posi.										3
	*C. jejuni*																				Posi.	1
Nakdong River	*S. sonnei*																					0
	*E. coli* O157		Posi.	Posi.	Posi.						Posi.	Posi.					Posi.					6
	*S. enterica*		Posi.	Posi.						Posi.												3
	*L. pneumophila*	Posi.	Posi.	Posi.																		3
	*V. cholerae*		Posi.	Posi.	Posi.							Posi.										4
	*C. jejuni*		Posi.																			1
	Total	6	20	13	10	1	1	0	4	7	7	3	0	0	0	0	2	0	1	0	2	77

**Table 6:** A statistical association between the monitored noxious bacteria and the tested indicator bacteria.

Noxious bacteria	Indicator bacteria	*p*-value	Bonferroni-adjusted *p*-value
*S. sonnei*	TC	0.148	1
	FC	0.304	1
	*E. coli*	0.38	1
*E. coli* 0157	TC	0.268	1
	FC	0.065	1
	*E. coli*	0.905	1
*S. enterica*	TC	0.014	0.252
	FC	0.262	1
	*E. coli*	0.28	1
*L. pneumophila*	TC	0.326	1
	FC	0.806	1
	*E. coli*	0.327	1
*V. cholerae*	TC	0.54	1
	FC	0.102	1
	*E. coli*	0.97	1
*C. jejuni*	TC	0.876	1
	FC	0.204	1
	*E. coli*	0.264	1
